# Outlines of Chemistry

**Published:** 1856-04

**Authors:** 


*Outlines of Chemistry for the use of Students.* By William Gregory, M. D., Prof. of Chemistry in the University of Edinburgh. Revised, corrected, and enlarged by J. Milton Sanders, M. D., &c. Published by A. S. Barnes & Co., New York.

As the author, in his preface, remarks, this work is designed for the use of students in attendance upon chemical lectures, and more particularly for those under his own instruction. The distinguished reputation of the author, as an accomplished chemist and teacher, is warrant sufficient of its worth, and its adaptation to the purpose for which it was designed, and more, of its value to the practical chemist.

For general use, however, in common or other schools, where the study of chemistry is pursued without aid from practical experiments, in our opinion, it is not so well suited as some other works. M.

